# Effects of *Trichoderma harzianum* and *Azospirillum brasilense* on tomato growth, fruit quality, yield, and water productivity under deficit irrigation

**DOI:** 10.1038/s41598-026-39498-0

**Published:** 2026-02-09

**Authors:** Ayoub Ghorbani Dehkordi, Kambiz Mashayekhi, Seyyed Javad Mousavizadeh, Kamran Rahnama

**Affiliations:** 1 Horticultural Sciences and Landscape Engineering Department, Gorgan University of Agricultural Sciences and Natural Resources, Gorgan, Iran; 2 Plant Protection Department, Gorgan University of Agricultural Sciences and Natural Resources, Gorgan, Iran

**Keywords:** Antioxidant, Biochemical characteristics, Biological fertilizers, Drought stress, Rainfed, Plant ecology, Plant physiology

## Abstract

This study evaluated tomato plants’ physiological and biochemical changes influenced by the *Trichoderma harzianum* and the *Azospirillum brasilense*. This study aimed to determine whether microbial inoculation with *T. harzianum* and *A. brasilense* can mitigate water-deficit stress and improve tomato growth, yield, and crop water productivity under different irrigation regimes. The irrigation regime was applied at four levels: no irrigation, 50%, 75%, and 100% of the water requirement (WR), and biological fertilizer was applied in four treatments: control, Trichoderma, Azospirillum, and Trichoderma + Azospirillum. The experiment was conducted as a factorial design, and the effects of irrigation regime and biological fertilizer treatments were statistically significant for most measured traits. The results demonstrated that the highest fresh and dry root weights and leaf area, were observed under the 100% WR combined with the application of biological fertilizers, particularly *Trichoderma*. Additionally, chlorophyll content was higher under a 100% WR with biological fertilizers. In contrast, The highest contents of carotenoids (22%), anthocyanins (18%), glucose (79%), sucrose (96%), and total sugars (121%) in leaves were observed under no-irrigation conditions. Conversely, the assessment of fruit characteristics revealed that the highest fresh and dry fruit weights, fresh fruit yield and Wp were achieved under the 100% WR combined with applying biological fertilizers, particularly *Trichoderma*. *Azospirillum* treatment and combining *Trichoderma* with *Azospirillum* achieved the lowest levels of fruit firmness, total soluble solids, and anthocyanin in fruits under a 100% WR. Moreover, the *Trichoderma* treatment was able to achieve similar performance under 75% WR conditions as the control treatment under 100% WR. Overall, the results indicate that Trichoderma inoculation plays a key role in improving tomato physiological performance and water productivity under different irrigation regimes, with potential implications for sustainable water management.

## Introduction

Tomatoes were produced globally in 2023, with approximately 192 million tons^1^. As one of the most important crops and commercially valuable vegetables worldwide, tomatoes are known for their diverse applications and high nutritional and medicinal value. Tomato cultivation has grown considerably in Iran and globally, with respective growth rates of 34% and 6.21% between 2000 and 2018 ^2^. Given the ongoing water crisis in Iran, implementing effective water management strategies tailored to the crop’s requirements is crucial for preserving water resources and mitigating scarcity. Accurately determining the plant’s water needs at various growth stages and ensuring their fulfillment can optimize water usage, prevent excessive consumption, and reduce the risk of deficit irrigation, which may negatively impact yields and quality.

Soil is a dynamic and complex ecosystem, hosting intricate interactions between living organisms and plants. Since chemical fertilizers may have negative environmental effects and threaten food safety and security, using organic products for the nutrition of agricultural and horticultural plants has been considered a fundamental solution^3^. In addition, organic products not only improve soil fertility but also promote environmental sustainability while contributing to increased agricultural output^4^.

Many studies have been conducted on microorganisms that can help plants absorb water and nutrients even in critical water stress conditions by establishing symbiotic relationships with plants. One key mechanism by which microorganisms support plants in tolerating water stress is their ability to modify the rhizosphere environment, thereby enhancing root absorption capacity. Another effect is stimulating the synthesis of antioxidant enzymes and improving plant resistance to stress conditions^5^.

*A. brasilense* is a well-known plant growth–promoting rhizobacterium (PGPR) widely used in tomato cultivation due to its capacity to enhance plant growth and nutrient availability, particularly under suboptimal environmental conditions^6,7^. The beneficial effects of *Azospirillum* are mediated through multiple physiological mechanisms, including biological nitrogen fixation, stimulation of root system architecture, and modulation of phytohormone signaling pathways such as auxin production^6,8,9^. These processes promote the formation of lateral roots and root hairs, thereby increasing root surface area and improving water and nutrient uptake under water-limited conditions^5,10^. In addition, *Azospirillum* has been reported to influence plant stress physiology by enhancing osmotic adjustment and regulating antioxidant activity, contributing to improved drought tolerance^10^. However, the magnitude of plant responses to *Azospirillum* inoculation is strongly influenced by environmental factors such as soil moisture, nutrient availability, and plant–microbe compatibility, highlighting the need for its evaluation under defined irrigation regimes^7^.

Beyond bacterial PGPRs, beneficial filamentous fungi such as *Trichoderma* spp. also play a key role in the rhizosphere by interacting directly with plant roots and modifying the soil–root interface^3,11^. *T. harzianum* is recognized as a multifunctional microbial inoculant that enhances plant performance through improved nutrient solubilization, stimulation of root growth, and activation of plant defense and stress-response pathways^3,12,13^. From a physiological perspective, *Trichoderma* can influence plant water relations by promoting root biomass development, enhancing root hydraulic conductivity, and modulating stomatal behavior, collectively contributing to improved water uptake and water-use efficiency under drought stress^14–17^. In addition, *T. harzianum* induces systemic resistance and enhances tolerance to both biotic and abiotic stresses, supporting plant performance under variable irrigation conditions^18–20^.

The current research aimed to evaluate the potential beneficial effects of *T. harzianum* and *A. brasilense* in increasing crop water productivity (WP), enhancing crop yield, and assessing the possibility of reducing irrigation without compromising tomato yield.

## Results

### Root fresh weight

Drought stress led to a reduction in root fresh weight across all treatments (Table 1). The highest root fresh weight was observed under the 100% WR (water requirement) condition with *Trichoderma* treatment. Compared to the control (100% WR with no biological fertilizer), root fresh weight increased by 2%, 10%, and 6% in the *Azospirillum*, *Trichoderma*, and *Trichoderma* + *Azospirillum* treatments, respectively, under the 100% WR condition. In the 75% WR condition, root fresh weight decreased by 6%, 4% and 7% in the *Azospirillum*, *Trichoderma* and *Trichoderma* + *Azospirillum* treatments, respectively. Similarly, under 50% WR, root fresh weight showed reductions of 9%, 14%, 16%, and 18% in the *Azospirillum*, *Trichoderma*, *Trichoderma* + *Azospirillum*, and no biological fertilizer treatments, respectively, compared to the control. Under the no irrigation condition, the decreases were more pronounced, with reductions of 12%, 20%, 22%, and 29% in the *Azospirillum*, *Trichoderma*, *Trichoderma* + *Azospirillum*, and no biological fertilizer treatments, respectively.

**Table 1:** Root dry and fresh weight and leaf area of tomato leaves at different irrigation regimes and biofertilizer treatments.

Irrigation	Biofertilizer	Root fresh weight		Root dry weight		Leaf Area	
		(g/plant)		(g/plant)		(m^2^/plant)	
Not-irrigated	Az	146.17	hi	11.08	lm	2.86	e-g
	Tr	132.83	kl	9.63	n	1.92	l
	Tr+Az	129.33	l	13.92	h	2.36	j
	Control	118.67	m	10.52	m	2.16	k
50% WR	Az	151	gh	11.87	kl	2.9	ef
	Tr	142.83	ij	14.2	gh	2.44	ij
	Tr+Az	138.67	jk	14.8	fg	2.68	gh
	Control	136.17	k	12.95	ij	2.28	jk
75% WR	Az	155.67	d-g	12.52	jk	2.99	de
	Tr	159	ef	15.2	ef	3.33	bc
	Tr+Az	153.83	fg	16.37	d	2.75	f-h
	Control	161.33	de	13.65	hi	2.62	hi
100% WR	Az	170	bc	20.48	a	3.58	a
	Tr	183.17	a	19	b	3.43	ab
	Tr+Az	175.83	b	17.82	c	3.14	cd
	Control	166	cd	15.97	de	3.03	de

### Root dry weight

The results indicated that the highest root dry weight was observed in the *Azospirillum* treatment under the 100% WR condition (Table 1). Compared to the control treatment (100% WR with no biological fertilizer), root dry weight increased by 28%, 19%, and 12% in the *Azospirillum*, *Trichoderma*, and *Trichoderma* + *Azospirillum* treatments, respectively, under 100% WR. In the 75% WR condition, root dry weight decreased by 22%, 5%, and 15% in the *Azospirillum*, *Trichoderma*, and *Trichoderma* + *Azospirillum* treatments, respectively. No biological fertilizer use resulted in a 3% increase compared to the control. Under the 50% WR condition, root dry weight decreased by 26%, 11%, 7%, and 19% in the *Azospirillum*, *Trichoderma*, *Trichoderma* + *Azospirillum*, and no biological fertilizer treatments, respectively. In the no irrigation condition, root dry weight showed 31%, 41%, 13%, and 34% reductions in the *Azospirillum*, *Trichoderma*, *Trichoderma* + *Azospirillum*, and no biological fertilizer treatments, respectively.

### Leaf area

Drought stress reduced leaf area, varying depending on the irrigation level and biological fertilizer treatments (Table 1). The highest leaf area was recorded under the 100% WR condition with *Azospirillum* treatment. In 100% WR treatment, leaf area showed an increase of 18%, 13%, and 4% in *Azospirillum*, *Trichoderma*, and *Trichoderma* + *Azospirillum* treatments, respectively, compared to the control treatment (100% WR without biological fertilizer use). Under the 75% WR, leaf area decreased by 1%, 10%, 9%, and 14% in *Azospirillum*, *Trichoderma*, *Trichoderma + Azospirillum*, and no biological fertilizer use treatments, respectively. Even in 50% WR, leaf area was reduced by 4%, 20%, 12%, and 25% in *Azospirillum*, *Trichoderma*, *Trichoderma + Azospirillum*, and no biological fertilizer use treatments, respectively. In no irrigation treatment, leaf area decreased by 6%, 37%, 22%, and 29% in *Azospirillum*, *Trichoderma*, *Trichoderma + Azospirillum*, and no biological fertilizer use treatments, respectively. Additionally, leaf area showed a direct and positive correlation with root fresh and dry weight.

### Chlorophyll a of leaves

Drought stress significantly reduced chlorophyll a content across treatments (Table 2). The highest chlorophyll a content was recorded under the 100% WR condition with *Azospirillum* treatment. Compared to the control treatment (100% WR without biological fertilizer), chlorophyll a content increased by 5% in the *Azospirillum* treatment but decreased by 3% and 5% in the *Trichoderma* and *Trichoderma* + *Azospirillum* treatments, respectively. Under the 75% WR condition, chlorophyll a decreased by 5%, 7%, 8%, and 10% in the *Azospirillum*, *Trichoderma*, *Trichoderma* + *Azospirillum*, and no biological fertilizer treatments, respectively. Similarly, in the 50% WR condition, 6%, 4%, 14%, and 12% reductions were observed in the *Azospirillum*, *Trichoderma*, *Trichoderma* + *Azospirillum*, and no biological fertilizer treatments, respectively. The no irrigation condition resulted in the most pronounced decreases, with chlorophyll a content reduced by 15%, 17%, 19%, and 8% in the *Azospirillum*, *Trichoderma*, *Trichoderma* + *Azospirillum*, and no biological fertilizer treatments, respectively.

**Table 2:** Content of pigments of tomato leaves at different irrigation regimes and biofertilizer treatments.

Irrigation	Biofertilizer	Chlorophyll a		Chlorophyll b		Total Chlorophyll		Carotenoids		Anthocyanin	
		(mg. g-1 FW)		(mg. g-1 FW)		(mg. g-1 FW)		(mg. g-1 FW)		(µg. g-1 FW)	
Not-irrigated	Az	131	lm	63	j	198	jk	71	C	1925	b
	Tr	128	mn	64	j	190	l	69	ef	1833	de
	Tr+Az	125	n	67	gh	194	k	74	b	1654	jk
	Control	141	hi	58	l	177	m	77	a	1543	l
50% WR	Az	144	e-g	68	ef	207	gh	69	de	1885	c
	Tr	148	cd	65	i	202	ij	63	j	1769	fg
	Tr+Az	133	j-l	66	hi	221	d	67	gh	1733	gh
	Control	136	jk	61	k	200	j	71	c	1691	ij
75% WR	Az	147	de	69	e	210	fg	68	fg	1974	a
	Tr	143	f-h	67	fg	212	ef	61	k	1859	cd
	Tr+Az	142	gh	76	a	231	b	66	hi	1756	g
	Control	138	ij	66	g-i	204	hi	70	cd	1617	k
100% WR	Az	162	a	71	cd	214	e	65	i	2010	a
	Tr	150	c	73	b	225	c	59	l	1873	c
	Tr+Az	146	d-f	72	bc	238	a	66	h	1802	ef
	Control	154	b	70	d	219	d	63	j	1707	hi

WR; water requirement, Az; *Azospirillum*, Tr; *Trichoderma*, Az+Tr; *Azospirillum*+ *Trichoderma*, FW; Fresh weight. Means with at least a common letter are not significantly different using the Least Significant Difference (LSD) test (*p-value* < 0.05).

### Chlorophyll b of leaves

Drought stress generally resulted in a reduction in chlorophyll b content across treatments (Table 2). The highest chlorophyll b content was observed under the 100% WR condition with *Azospirillum* treatment. Compared to the control treatment (100% WR without biological fertilizer), chlorophyll b content increased by 1%, 4%, and 3% in the *Azospirillum*, *Trichoderma*, and *Trichoderma* + *Azospirillum* treatments, respectively, under 100% WR. In the 75% WR condition, chlorophyll b content decreased by 1%, 4%, and 6% in the *Azospirillum*, *Trichoderma*, and no biological fertilizer treatments. A 9% increase was observed in the *Trichoderma* + *Azospirillum* treatment compared to the control. Under 50% WR, chlorophyll b decreased by 3%, 7%, 6%, and 13% in the *Azospirillum*, *Trichoderma*, *Trichoderma* + *Azospirillum*, and no biological fertilizer treatments, respectively. In the no irrigation condition, the decreases were 10%, 9%, 4%, and 17% in the *Azospirillum*, *Trichoderma*, *Trichoderma* + *Azospirillum*, and no biological fertilizer treatments, respectively.

### Total chlorophyll of leaves

Drought stress resulted in a reduction in total chlorophyll content across treatments (Table 2). The total chlorophyll content was recorded under the 75% WR condition with the *Trichoderma* + *Azospirillum* treatment. Under 100% WR, total chlorophyll content decreased by 2% in the *Azospirillum* treatment but increased by 3% and 9% in the *Trichoderma* and *Trichoderma* + *Azospirillum* treatments, respectively, compared to the control treatment (100% WR without biological fertilizer). In the 75% WR condition, total chlorophyll content decreased by 4%, 3%, and 7% in the *Azospirillum*, *Trichoderma*, and no biological fertilizer treatments, respectively, while a 5% increase was observed in the *Trichoderma* + *Azospirillum* treatment. Under the 50% WR condition, total chlorophyll content decreased by 5%, 8%, and 9% in the *Azospirillum*, *Trichoderma*, and no biological fertilizer treatments, respectively, whereas the *Trichoderma* + *Azospirillum* treatment showed a 1% increase compared to the control. In the no irrigation condition, total chlorophyll content decreased by 10%, 13%, 11%, and 19% in the *Azospirillum*, *Trichoderma*, *Trichoderma* + *Azospirillum*, and no biological fertilizer treatments, respectively.

### Carotenoid content of leaves

The highest leaf carotenoid content was observed in the no biological fertilizer treatment under no irrigation conditions (Table 2). Under 100% WR conditions, carotenoid content increased by 3% and 5% in the *Azospirillum* and *Trichoderma* + *Azospirillum* treatments, respectively, compared to the control treatment (100% WR without biological fertilizer). However, a 6% decrease in carotenoid content was noted in the *Trichoderma* treatment compared to the control. In the 75% WR condition, carotenoid content increased by 8%, 5%, and 11% in the *Azospirillum*, *Trichoderma* + *Azospirillum*, and no biological fertilizer treatments, respectively, while a 3% decrease was observed in the *Trichoderma* treatment. Under the 50% WR condition, carotenoid content decreased by 10%, 6%, and 13% in the *Azospirillum*, *Trichoderma* + *Azospirillum*, and no biological fertilizer treatments, respectively, with no significant change observed in the *Trichoderma* treatment. In the no irrigation condition, carotenoid content increased by 13%, 10%, 17%, and 22% in the *Azospirillum*, *Trichoderma*, *Trichoderma* + *Azospirillum*, and no biological fertilizer treatments, respectively.

### Anthocyanin content of leaves

Drought stress generally led to increased anthocyanin content in leaves, varying depending on the irrigation level and the application of biological fertilizers (Table 2). The highest anthocyanin content was observed without biological fertilizers under rainfed conditions. Under 100% WR, anthocyanin content decreased by 10%, 1%, and 5% in the *Azospirillum*, *Trichoderma*, and *Trichoderma* + *Azospirillum* treatments, respectively, compared to the control treatment (100% WR without biological fertilizer). In the 75% WR condition, anthocyanin content decreased by 3% in the *Azospirillum* treatment but increased by 2%, 3%, and 6% in the *Trichoderma*, *Trichoderma* + *Azospirillum*, and no biological fertilizer treatments, respectively. Under 50% WR, anthocyanin content increased by 7%, 4%, 9%, and 10% in the *Azospirillum*, *Trichoderma*, *Trichoderma* + *Azospirillum*, and no biological fertilizer treatments, respectively. In the no irrigation condition, anthocyanin content increased further by 13%, 10%, 16%, and 18% in the *Azospirillum*, *Trichoderma*, *Trichoderma* + *Azospirillum*, and no biological fertilizer treatments, respectively. Additionally, a significant positive correlation was observed between anthocyanin content and total soluble solids, fruit firmness, and leaf carotenoids. In contrast, significant negative correlations were noted between anthocyanin content and traits such as root fresh and dry weight, leaf area, fruit fresh and dry weight, and total leaf chlorophyll content.

### Glucose of leaves

**Fig. 1:**
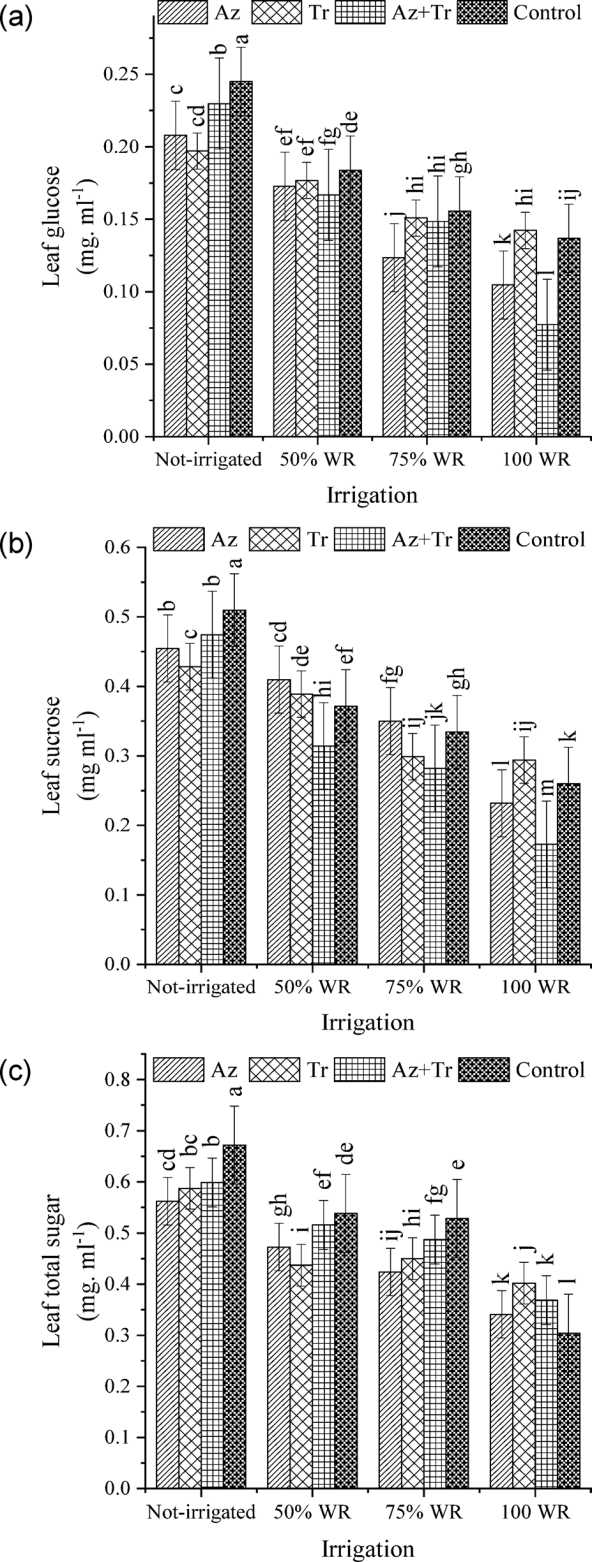
Content of glucose (**a**), sucrose (**b**) and total sugar (**c**) of tomato leaves at different irrigation regimes and biofertilizer treatments (WR; water requirement, Az; *Azospirillum*, Tr; *Trichoderma*, Az + Tr; *Azospirillum*+ *Trichoderma*). Means with at least a common letter are not significantly different using the Least Significant Difference (LSD) test (p-value < 0.05).

The drought stress led to an increase in leaf glucose (Fig. 1a), and the highest leaf glucose was obtained from no biofertilizer consumption under no irrigation conditions. Under 100% WR, leaf glucose content decreased by 24% in the *Azospirillum* treatment, increased by 4% in the *Trichoderma* treatment, and decreased by 43% in the *Trichoderma* + *Azospirillum* treatment, compared to the control (100% WR without biological fertilizer). In the 75% WR condition, leaf glucose content decreased by 10% in the *Azospirillum* treatment but increased by 10%, 9%, and 14% in the *Trichoderma*, *Trichoderma* + *Azospirillum*, and no biological fertilizer treatments, respectively. Under the 50% WR condition, leaf glucose content increased by 26%, 29%, 22%, and 34% in the *Azospirillum*, *Trichoderma*, *Trichoderma* + *Azospirillum*, and no biological fertilizer treatments, respectively. In the no irrigation condition, leaf glucose content showed further increases of 52%, 44%, 68%, and 79% in the *Azospirillum*, *Trichoderma*, *Trichoderma* + *Azospirillum*, and no biological fertilizer treatments.

### Sucrose of leaves

Drought stress increased leaf sucrose (Fig. 1b), and the highest leaf sucrose was obtained from no biofertilizer treatment in no irrigation conditions. Under 100% WR, leaf sucrose content decreased by 11% in the *Azospirillum* treatment, increased by 13% in the *Trichoderma* treatment, and decreased by 34% in the *Trichoderma* + *Azospirillum* treatment compared to the control (100% WR without biological fertilizer). In the 75% WR condition, leaf sucrose content increased by 35%, 15%, 8%, and 29% in the *Azospirillum*, *Trichoderma*, *Trichoderma* + *Azospirillum*, and no biological fertilizer treatments, respectively. Under the 50% WR condition, leaf sucrose content further increased by 58%, 50%, 21%, and 43% in the *Azospirillum*, *Trichoderma*, *Trichoderma* + *Azospirillum*, and no biological fertilizer treatments, respectively. The no irrigation condition showed the most pronounced increases in leaf sucrose, with increments of 75%, 65%, 83%, and 96% in the *Azospirillum*, *Trichoderma*, *Trichoderma* + *Azospirillum*, and no biological fertilizer treatments, respectively.

### Total leaf sugar

The results indicated that drought stress increased total leaf sugar content, with the magnitude of the increase varying across irrigation levels and biological fertilizer treatments (Fig. 1c). The highest total leaf sugar content was observed in the no biological fertilizer treatment under no irrigation conditions. Under 100% WR, total leaf sugar content increased by 12%, 32%, and 21% in the *Azospirillum*, *Trichoderma*, and *Trichoderma* + *Azospirillum* treatments, respectively, compared to the control (100% WR without biological fertilizer). In the 75% WR condition, total leaf sugar content increased by 39%, 48%, 60%, and 74% in the *Azospirillum*, *Trichoderma*, *Trichoderma* + *Azospirillum*, and no biological fertilizer treatments, respectively. Under the 50% WR condition, total leaf sugar content increased further by 55%, 44%, 70%, and 77% in the *Azospirillum*, *Trichoderma*, *Trichoderma* + *Azospirillum*, and no biological fertilizer treatments, respectively. The no irrigation condition showed the most substantial increases, with total leaf sugar content rising by 85%, 93%, 97%, and 121% in the *Azospirillum*, *Trichoderma*, *Trichoderma* + *Azospirillum*, and no biological fertilizer treatments, respectively.

### Fruit fresh weight

**Fig. 2:**
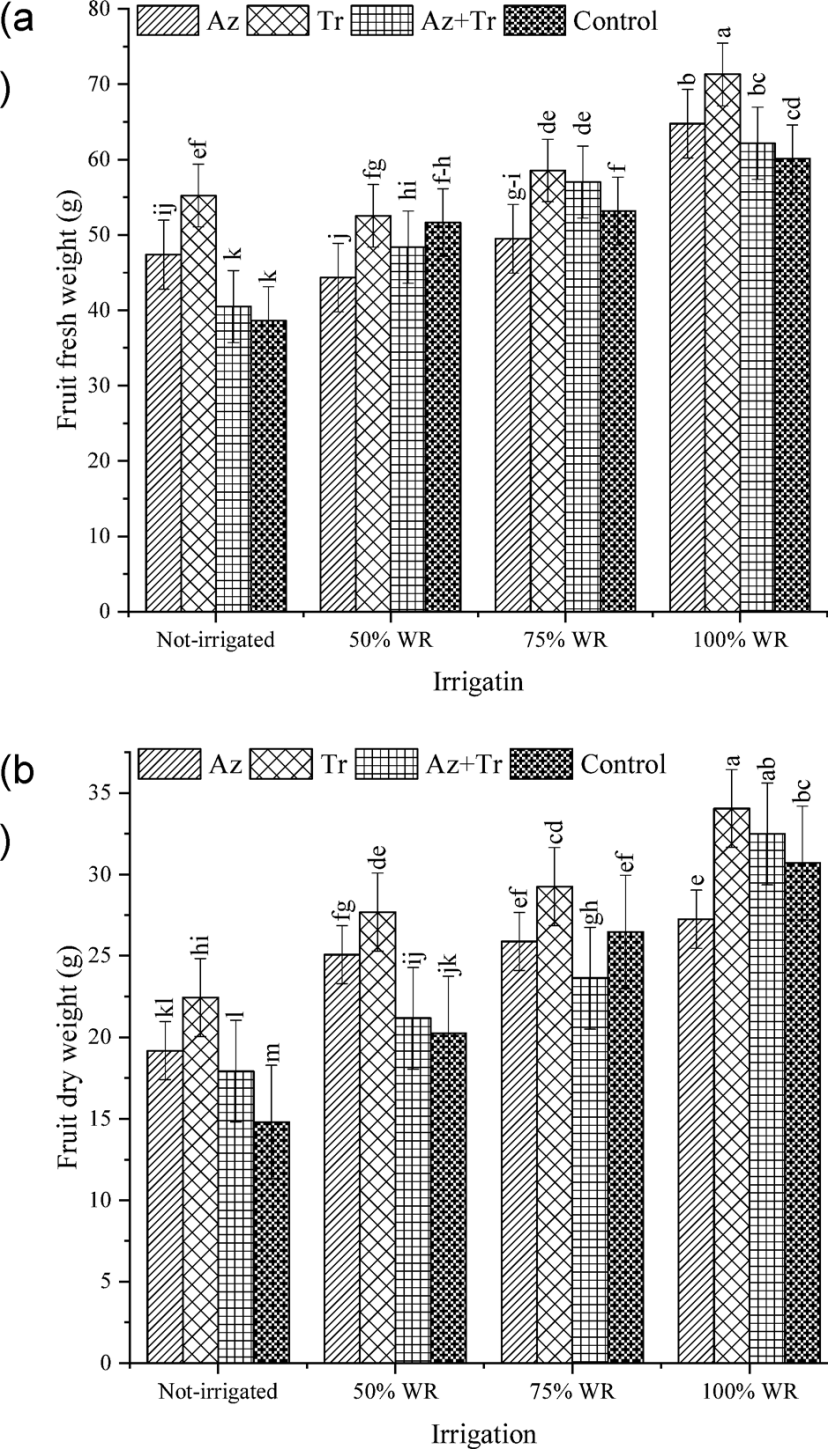
Fruit fresh (**a**) and dry (**b**) weight of tomato at different irrigation regimes and biofertilizer treatments (WR; water requirement, Az; *Azospirillum*, Tr; *Trichoderma*, Az + Tr; *Azospirillum*+ *Trichoderma*). Means with at least a common letter are not significantly different using the Least Significant Difference (LSD) test (p-value < 0.05).

The results revealed that drought stress caused a decrease in fruit fresh weight, with the extent of reduction varying across irrigation levels and biological fertilizer treatments (Fig. 2a). The highest fresh fruit weight was recorded in the *Trichoderma* treatment under 100% WR conditions. Under 100% WR, fruit fresh weight increased by 8%, 19%, and 3% in the *Azospirillum*, *Trichoderma*, and *Trichoderma* + *Azospirillum* treatments, respectively, compared to the control (100% WR without biological fertilizer). In the 75% WR condition, fruit fresh weight decreased by 18%, 3%, 5%, and 12% in the *Azospirillum*, *Trichoderma*, *Trichoderma* + *Azospirillum*, and no biological fertilizer treatments, respectively. Under 50% WR, the decreases were more pronounced, with reductions of 26%, 13%, 20%, and 14% in the *Azospirillum*, *Trichoderma*, *Trichoderma* + *Azospirillum*, and no biological fertilizer treatments, respectively. In the no irrigation condition, fruit fresh weight decreased by 21%, 8%, 33%, and 36% in the *Azospirillum*, *Trichoderma*, *Trichoderma* + *Azospirillum*, and no biological fertilizer treatments, respectively.

### Fruit dry weight

The results demonstrated that drought stress reduced fruit dry weight, with the severity of the decrease varying based on irrigation levels and biological fertilizer treatments (Fig. 2b). The highest fruit dry weight was observed in the *Trichoderma* treatment under 100% WR conditions. Under 100% WR, fruit dry weight decreased by 11% in the *Azospirillum* treatment but increased by 11% and 6% in the *Trichoderma* and *Trichoderma* + *Azospirillum* treatments, respectively, compared to the control (100% WR without biological fertilizer). In the 75% WR condition, fruit dry weight decreased by 16%, 5%, 23%, and 14% in the *Azospirillum*, *Trichoderma*, *Trichoderma* + *Azospirillum*, and no biological fertilizer treatments, respectively. Under the 50% WR condition, 18%, 10%, 31%, and 34% reductions were observed in the *Azospirillum*, *Trichoderma*, *Trichoderma* + *Azospirillum*, and no biological fertilizer treatments. In the no irrigation condition, the decreases were most pronounced, with fruit dry weight reductions of 38%, 27%, 42%, and 52% in the *Azospirillum*, *Trichoderma*, *Trichoderma* + *Azospirillum*, and no biological fertilizer treatments, respectively.

### Fruit firmness

**Fig. 3:**
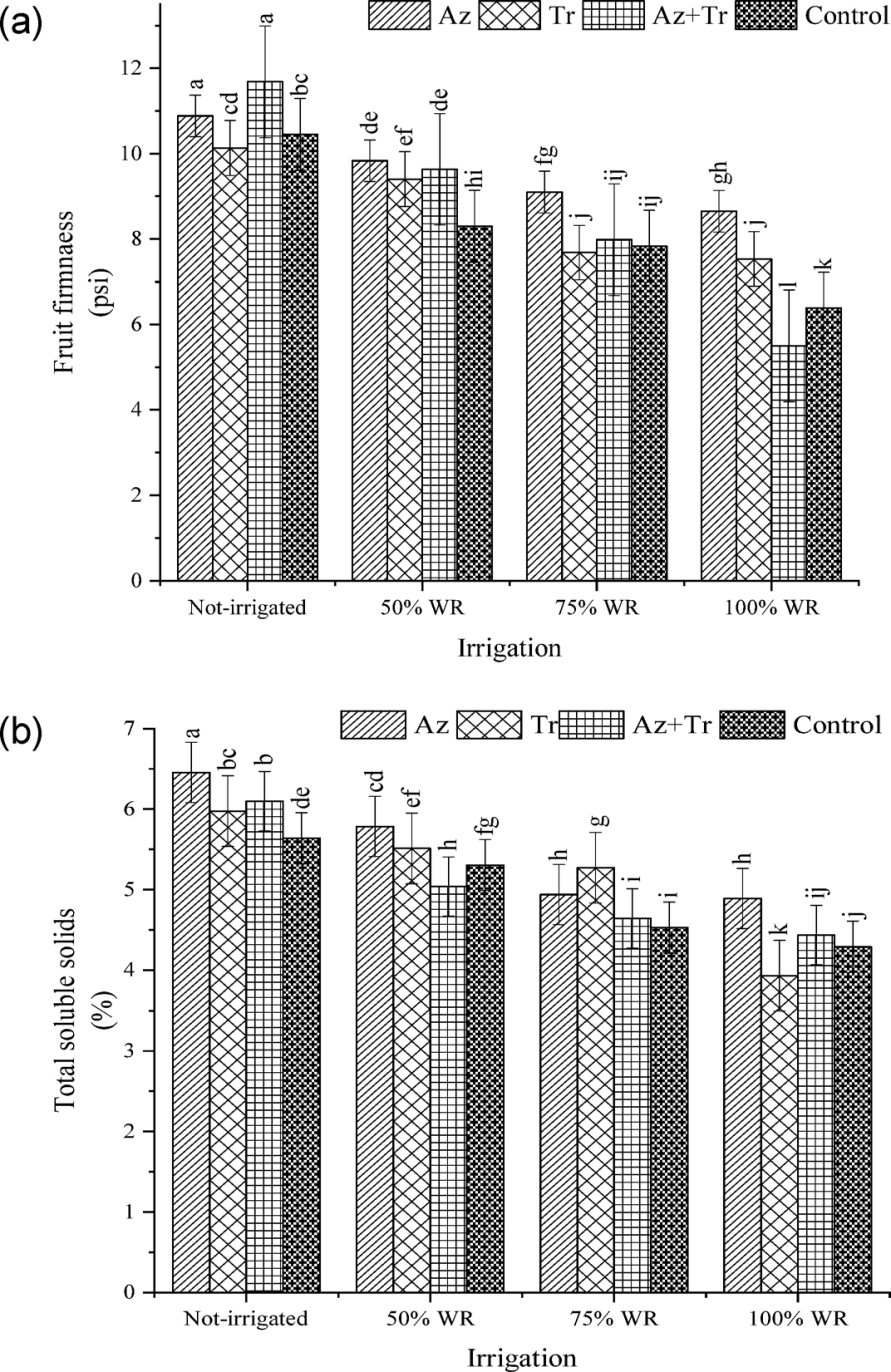
Fruit firmness (**a**) and total soluble solids (**b**) of tomato fruits at different irrigation regimes and biofertilizer treatments (WR; water requirement, Az; *Azospirillum*, Tr; *Trichoderma*, Az+Tr; *Azospirillum*+ *Trichoderma*). Means with at least a common letter are not significantly different using the Least Significant Difference (LSD) test (p-value < 0.05).

The results indicated that drought stress increased fruit firmness (Fig. 3a). The most fruit firmness was obtained from *Trichoderma+Azospirillum* treatment in no irrigation conditions. Under 100% WR, fruit firmness increased by 36% and 18% in the *Azospirillum* and *Trichoderma* treatments, respectively, but decreased by 14% in the *Trichoderma* + *Azospirillum* treatment compared to the control (100% WR without biological fertilizer). In the 75% WR condition, fruit firmness increased by 43%, 20%, 25%, and 23% in the *Azospirillum*, *Trichoderma*, *Trichoderma* + *Azospirillum*, and no biological fertilizer treatments, respectively. Under the 50% WR condition, increases of 54%, 47%, 51%, and 30% were observed in the *Azospirillum*, *Trichoderma*, *Trichoderma* + *Azospirillum*, and no biological fertilizer treatments, respectively. In the no irrigation condition, fruit firmness showed the highest increases, with increments of 70%, 59%, 83%, and 64% in the *Azospirillum*, *Trichoderma*, *Trichoderma* + *Azospirillum*, and no biological fertilizer treatments, respectively.

### Fruit total soluble solids

The results showed that drought stress increased the total soluble solid content of the fruit (Fig. 3b), with the highest content observed in the *Azospirillum* treatment under no irrigation conditions. In 100% WR, the soluble solid content increased by 14% in the *Azospirillum* treatment but decreased by 8% and 3% in the *Trichoderma* and *Trichoderma* + *Azospirillum* treatments, respectively, compared to the control (100% WR with no biological fertilizer). Under 75% WR, the soluble solid content increased by 15%, 23%, 8%, and 6% in the *Azospirillum*, *Trichoderma*, *Trichoderma* + *Azospirillum*, and no biological fertilizer treatments, respectively. In the 50% WR treatment, the increases were 35%, 29%, 17%, and 24% in the *Azospirillum*, *Trichoderma*, *Trichoderma* + *Azospirillum*, and no biological fertilizer treatments, respectively. Even under no irrigation conditions, the total soluble solid content of the fruit increased by 50%, 39%, 42%, and 31% in the *Azospirillum*, *Trichoderma*, *Trichoderma* + *Azospirillum*, and no biological fertilizer treatments, respectively.

### Fresh fruit yield

**Fig. 4:**
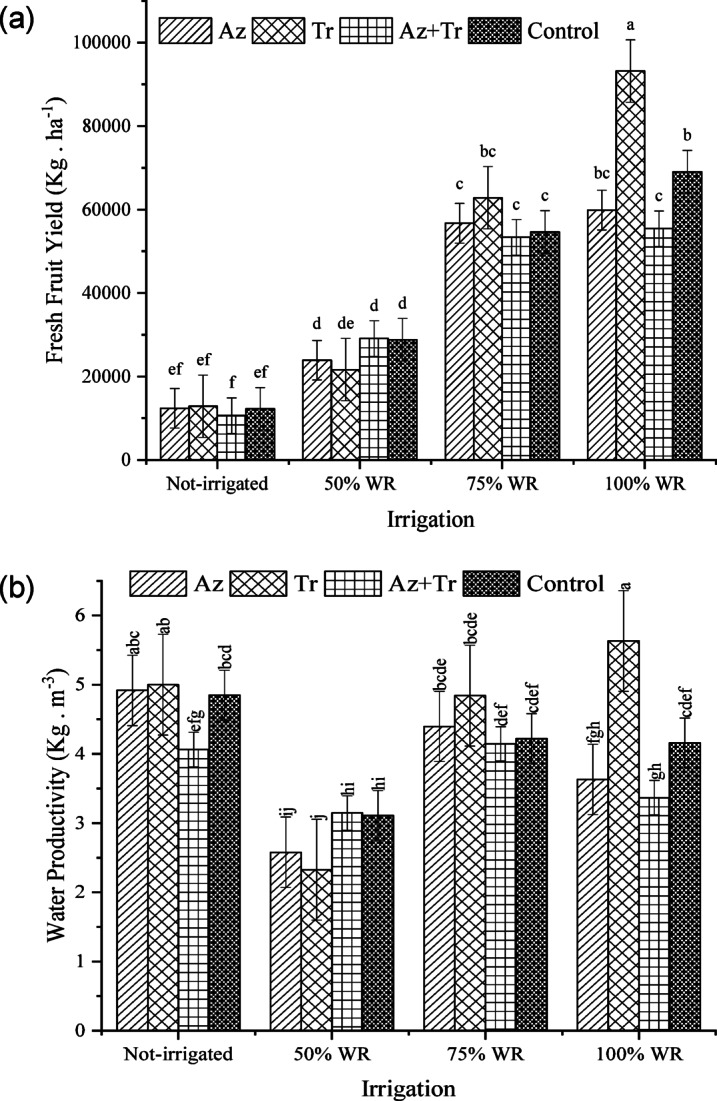
Fresh fruit yield (**a**) and crop water productivity (**b**) at different irrigation regimes and biofertilizer treatments (WR; water requirement, Az; *Azospirillum*, Tr; *Trichoderma*, Az + Tr; *Azospirillum*+ *Trichoderma*). Means with at least a common letter are not significantly different using the Least Significant Difference (LSD) test (p-value < 0.05).

The results indicated that tomato yield under the 100% WR was higher than under the other irrigation treatments (Fig. 4a). Within this regime, the highest yield was obtained with the application of the biological fertilizer *Trichoderma*, which was approximately 30% higher than the control. No significant difference was observed between the control and *Azospirillum* treatments. The combination treatment of *Trichoderma* + *Azospirillum* resulted in a lower performance. Under the 75%, 50% WR and no irrigation condition, no significant differences were found among the treatments, although under the 75% WR, the *Trichoderma* treatment performed relatively better than the others. On the other hand, the performance under the 75% WR combined with Trichoderma application did not show a significant difference compared to the control treatment under the 100% WR.

The correlation analysis revealed that total chlorophyll content was positively and directly correlated with fruit fresh weight (*r* = 0.69^**^), fruit dry weight (*r* = 0.73^**^), leaf area (*r* = 0.68^**^), root fresh weight (*r* = 0.79^**^) and root dry weight (*r* = 0.76^**^). In contrast, fruit firmness (*r*=-0.75^***^) and total soluble solids (*r*=-0.74^**^) content exhibited a significant negative correlation with total chlorophyll content. Furthermore, glucose, sucrose, and total leaf sugar were significantly correlated with anthocyanin levels (respectively *r* = 0.95^***^, *r* = 0.88^***^, *r* = 0.91^**^), carotenoid content (respectively *r* = 0.67^**^, *r* = 0.71^**^, *r* = 0.80^***^), fruit firmness (respectively *r* = 0.81^***^, *r* = 0.88^***^, *r* = 0.73^**^), and total soluble solids (respectively *r* = 0.74^**^, *r* = 0.81^***^, *r* = 0.68^**^). However, these sugars showed a significant negative correlation with total chlorophyll content (respectively *r*=-0.84^***^, *r*=-0.90^***^, *r*=-0.75^***^), fruit fresh weight (respectively *r*=-0.75^***^, *r*=-0.81^***^, *r*=-0.75^***^), fruit dry weight (respectively *r*=-0.82^***^, *r*=-0.79^***^, *r*=-0.87^***^), leaf area (respectively *r*=-0.74^**^, *r*=-0.71^**^, *r*=-0.79^***^), root fresh weight (respectively *r*=-0.86^***^, *r*=-0.83^***^, *r*=-0.85^***^) and root dry weight (respectively *r*=-0.73^**^, *r*=-0.83^***^, *r*=-0.75^***^). Furthermore, a significant positive correlation was observed between yield and root fresh weight (*r* = 0.89^***^), root dry weight (*r* = 0.76^***^), leaf area (*r* = 0.78^***^), fruit fresh weight (*r* = 0.83^***^), fruit dry weight (*r* = 0.83^***^), the contents of chlorophyll a (*r* = 0.69^**^), chlorophyll b (*r* = 0.71^**^), and total chlorophyll (*r* = 0.72^**^). In contrast, a significant negative correlation was found between yield and total soluble solid content (*r*=-0.90^***^), fruit firmness (*r*=-0.79^***^), carotenoid content (*r*=-0.76^***^), anthocyanins (*r*=-0.79^***^), glucose (*r*=-0.77^***^), sucrose (*r*=-0.79^***^), and total sugars (*r*=-0.79^***^) (Table 3).

**Table 3:** Pearson correlation coefficients among measured traits.

Trait	Root FW	Root DW	LA	Fruit FW	Fruit DW	TSS	Fruit firmness.	Ch a	Ch b	Ch T	Car	Ant	Glu	Suc	Total Sugar
Root DW	0.77^***^														
LA	0.86^***^	0.77^***^													
Fruit FW	0.85^***^	0.77^***^	0.65^**^												
Fruit DW	0.91^***^	0.68^**^	0.71^**^	0.84^***^											
TSS	-0.76^***^	-0.73^**^	-0.55^*^	-0.73^**^	-0.74^**^										
Fruit firmness.	-0.77^***^	-0.62^**^	-0.54^*^	-0.75^***^	-0.80^***^	0.85^***^									
Ch a	0.68^**^	0.67^**^	0.71^**^	0.61^*^	0.67^**^	-0.63^**^	-0.59^*^								
Ch a	0.77^***^	0.75^***^	0.67^**^	0.66^**^	0.69^**^	-0.65^**^	-0.55^*^	0.59^*^							
Ch T	0.79^***^	0.76^***^	0.68^**^	0.69^**^	0.73^**^	-0.74^**^	-0.75^***^	0.56^*^	0.86^***^						
Car	-0.76^***^	-0.67^**^	-0.68^**^	-0.84^***^	-0.88^***^	0.61^*^	0.63^**^	-0.60^*^	-0.65^**^	-0.70^**^					
Ant	-0.86^***^	-0.77^***^	-0.79^***^	-0.80^***^	-0.84^***^	0.70^**^	0.72^**^	-0.82^***^	-0.73^**^	-0.74^**^	0.74^**^				
Glu	-0.86^***^	-0.73^**^	-0.74^**^	-0.75^***^	-0.82^***^	0.74^**^	0.81^***^	-0.69^**^	-0.74^**^	-0.84^***^	0.67^**^	0.95^***^			
Suc	-0.83^***^	-0.83^***^	-0.71^**^	-0.81^***^	-0.79^***^	0.81^***^	0.88^***^	-0.63^**^	-0.74^**^	-0.90^***^	0.71^**^	0.88^***^	0.94^***^		
Total Sugar	-0.85^***^	-0.75^***^	-0.79^***^	-0.75^***^	-0.87^***^	0.68^**^	0.73^**^	-0.82^***^	-0.70^**^	-0.75^***^	0.80^***^	0.91^***^	0.87^***^	0.84^***^	
Fresh fruit yield	0.89^***^	0.76^***^	0.78^***^	0.83^***^	0.83^***^	-0.90^***^	-0.79^***^	0.69^**^	0.71^**^	0.72^**^	-0.76^***^	-0.79^***^	-0.77^***^	-0.79^***^	-0.76^***^

Root FW; root fresh weight, Root DW; root dry weight, LA; leaf area, Fruit FW; fruit fresh weight, Fruit DW; fruit dry weight, TSS; total soluble solids, Ch a; chlorophyll a, Ch b; chlorophyll b, Ch T; total chlorophyll, Car; carotenoids, Ant; anthocyanin, Glu; glucose, Suc; sucrose. *, **, and *** indicate significant levels of *p-value* < 0.05, *p-value* < 0.01, and *p-value* < 0.001, respectively.

### Crop water productivity

The results showed that the highest Crop water productivity (WP) was achieved under the 100% WR with the *Trichoderma* treatment, where approximately 180 L of water were consumed per kilogram of tomato produced (Fig. 4b). No significant difference was observed between the control and the biological fertilizer *Azospirillum*, with 238 and 263 L of water used per kilogram of tomato, respectively. The lowest WP was found in the combined treatment of *Trichoderma* and *Azospirillum*, where 294 L of water were consumed per kilogram of tomato produced. No significant differences were observed among the treatments under the 75%, 50% WR and no irrigation condition. In the 50% WR, the WP was poorer compared to the other irrigation treatments, with water consumption ranging from 310 to 420 L per kilogram of tomato. According to the obtained results, the highest WP was observed in the *Trichoderma* treatment under the 100% WR. However, under non-irrigated conditions, the treatments with *Azospirillum* and the combination of *Trichoderma* + *Azospirillum* showed no significant difference compared to this treatment. Under 100% WR, the control treatment exhibited higher WP compared to the plants treated with *Azospirillum* and the *Trichoderma* + *Azospirillum* combination. No significant differences were observed among all treatments under non-irrigated conditions and those treated with *Azospirillum* and *Trichoderma* under the 75% WR. However, WP under the 50 WR was notably low.

## Discussion

The experimental findings demonstrated that biological compounds significantly enhanced plant physiological parameters across all WR. Under the 75% WR, *Azospirillum* application increased root dry weight, while *Trichoderma* and combined *Trichoderma*+*Azospirillum* treatments improved root fresh weight. Leaf area expansion under *Azospirillum* treatment exhibited no statistically significant difference from the control at 100% WR. Notably, *Trichoderma* application under 75% WR resulted in leaf area exceeding those observed in the 100% WR with the control treatment. These results underscore the pivotal role of biological compounds in stress mitigation strategies for tomato cultivation. The compounds enhance plant resilience through three primary mechanisms: cellular homeostasis maintenance, enhanced nutrient acquisition efficiency and improved stress response modulation. This multi-mechanismic action suggests biological compounds could serve as sustainable alternatives for maintaining crop productivity under water-limited conditions.

In the present study, plant inoculation with *Azospirillum* and *Trichoderma* improved root weight, especially under drought-stress conditions. Similarly, *Azospirillum* and *Trichoderma* have been reported to enhance root growth in Oliveira et al.^21^. Numerous reports have suggested the participation of *Azospirillum* in biological nitrogen fixation. For example, ^2121^ discussed biological nitrogen fixation by *Azospirillum*. This report focused on five crop plants and their assistance through biological nitrogen fixation. ^6 6^ inoculated the Brachiaria plant with a *A. brasilense* observed that the dry weight of roots and shoots and the nitrogen content were higher compared to non-inoculated plants.

When plants are inoculated with bacteria, the root system is typically the first to exhibit a response. Inoculation with beneficial microbes can increase root hair density and length, the number and length of lateral roots, root diameter, and overall root system area^21^. This enhanced root development improves the plant’s ability to acquire nutrients, ultimately contributing to better overall growth. The changes observed in the root system are often attributed to the production of auxins, with *Azospirillum* being particularly noted for its effectiveness^22^. For instance, Moreira et al.^23^ inoculated rice plants grown in a hydroponic medium with sterilized root exudates from *Azospirillum*. The inoculated plants exhibited increased root dry weight, greater root surface area, higher lateral roots, more root hairs, and increased root length compared to the non-inoculated controls.

*Trichoderma* inoculation influences the root system structure in tomato plants, which is crucial for enhancing the performance of dependent plants by increasing root biomass production and expanding hairy roots^12^. Moreover, *Trichoderma* stimulated tomato roots’ primary root elongation and branching by inducing lateral root growth^12,21^. A substantial increase in the height, diameter, and both fresh and dry weight of the aerial parts, along with higher concentrations of calcium, magnesium, phosphorus, and potassium in the stems and roots, was observed in tomato seedlings grown in *Trichoderma*-inoculated soil^24^. In addition, inoculation of tomato and lettuce plants with *Trichoderma* fungi significantly improved the weight of the aerial parts, likely due to *Trichoderma*’s ability to enhance water absorption and nutrient uptake^23^. Yedidia et al.^19^ demonstrated that soil treatment with *T. harzianum* strain resulted in cucumber seed germination increment by 30% eight days after planting, root area, and length enhancement by 95% and 75% respectively 28 days after planting, and leaf area enhanced by 80%.

*Trichoderma* inoculation may enhance nutrient uptake in tomato plants through multiple, interconnected mechanisms that directly influence plant growth and physiological performance. One of the primary effects of *Trichoderma* is the stimulation of root system development, including increased root biomass and root surface area, which improves the plant’s capacity to explore soil resources and absorb water and nutrients more efficiently. In addition, *Trichoderma* species are known to improve nutrient availability in the rhizosphere by solubilizing mineral nutrients and enhancing their accessibility to plant roots. These effects are particularly important under water-limited conditions, where nutrient diffusion in the soil is restricted. In the present study, the observed increases in root fresh and dry weight, leaf area, and chlorophyll content in *Trichoderma*-treated plants support this mechanism and suggest that improved nutrient uptake contributed to enhanced photosynthetic capacity and biomass accumulation. Similar improvements in plant growth and development following *Trichoderma* inoculation have been reported in previous studies, further confirming the role of this fungus in promoting nutrient acquisition and overall plant performance^11,14,24^.

*Trichoderma* strains produce growth factors such as auxins, cytokinins, ethylene, and cytokinin-like molecules such as zeatin and gibberellins, leading to increased root growth and plant development^25^. In addition, *Trichoderma* fungi exhibit different reactions in various conditions, including the presence and absence of different elements^14^. In other words, applying *Trichoderma* increases nutrient solubility in the soil, which may be among the reasons for improved growth in tomato plants inoculated with *Trichoderma* fungi. In Tucci et al.^26^, an increase in the tomato seedlings’ growth treated with *Trichoderma* fungi was reported due to enhanced growth and increased nitrogen absorption efficiency compared to untreated plants.

Many studies have highlighted the benefits of using *Azospirillum* in combination with other plant growth–promoting rhizobacteria (PGPRs) to mitigate the adverse effects of abiotic stresses, including salinity and water deficit^18,23^. The positive effects of *Azospirillum* under stress conditions are largely attributed to its ability to stimulate root growth, enhance phytohormone production—particularly auxins—and improve nutrient and water uptake efficiency^9,10^. By promoting lateral root formation and maintaining leaf expansion, *Azospirillum* inoculation can help sustain leaf surface area, which is critical for preserving photosynthetic capacity under stress conditions^5,10^. In the present study, the partial maintenance of leaf area and root biomass observed in *Azospirillum* brasilense–treated plants under reduced irrigation supports these mechanisms. However, the magnitude of this response was generally lower than that observed for *Trichoderma*, suggesting that while *Azospirillum* contributes to stress mitigation primarily through enhanced vegetative growth, its effect on overall water-use efficiency may be more limited under severe water deficit conditions.

A study by Ghorbanpour et al.^20^ demonstrated that tomato seedlings treated with *Trichoderma* fungus exhibited improved growth, particularly in height and leaf area, under cold and normal conditions. This improvement was attributed to the enhanced root ability to absorb soluble nutrients (in the first stage) and transfer them to different parts of the plant (in the second stage). Similar effects were observed in our experiment, where *Trichoderma* treatment promoted the growth of tomato plants. Additionally, the results from Ghorbanpour et al.^20^ indicated that increased fresh and dry root weight led to enhanced nutrient uptake, growth, and, consequently, more excellent leaf area development in the plant—findings that align with the results observed in our study.

This result highlights the impact of biological treatments on stress reduction in plants. The increased chlorophyll levels observed in the biological treatments enhanced the plants’ photosynthetic capacity. Conversely, the decrease in carotenoid levels indicated an improved tolerance to stress conditions. It is well established that drought stress causes chloroplast degradation and reduces the synthesis of chlorophyll a, chlorophyll b, and carotenoids, which alters the chlorophyll ratio, with chlorophyll b potentially being more resilient than chlorophyll a ^27^. Chloroplast destruction and chlorophyll breakdown, driven by enzymes such as chlorophyllase and peroxidase, contribute to the decreased pigment concentrations in photosynthetic tissues under water stress^28^. As a result, enhancing chlorophyll levels can increase photosynthetic efficiency, boost the production of photosynthetic materials, and potentially improve yields in cultivated plants. Additionally, the metabolic processes of the host plant are influenced by *Trichoderma* fungi^15^, which help the plant withstand environmental stresses and maintain growth under optimal conditions. In this experiment, the presence of *Trichoderma* in the rhizosphere of infected treatments likely facilitated the continuation of photosynthesis by preventing a significant decrease in photosynthetic pigments. Furthermore, researchers have linked the decline in chlorophyll content to nitrogen levels^11,14,29^. Therefore, establishing a symbiotic relationship in this study, which provided additional nitrogen to the aerial parts of the plant, especially the leaves, likely contributed to improved chlorophyll content, both under drought stress and control conditions.

In the current study, plant inoculation with *Azospirillum* improved anthocyanin levels in tomato leaves, particularly under 100% WR conditions. Increased anthocyanin accumulation in response to drought stress has been reported in previous studies^10^. Anthocyanins protect leaves from sunlight-induced damage, thus helping reduce oxidative stress in plants^10^. However, a consistent trend regarding the impact of growth-promoting microorganisms on anthocyanin concentration has not been universally observed. This study found no clear pattern in the effect of *Azospirillum* and *Trichoderma* application on anthocyanin concentration. This result is consistent with findings from Vukelić et al.^11^, who reported a significant increase in anthocyanin levels in tomato leaves treated with *Trichoderma* compared to control plants. Furthermore, it was noted that anthocyanin concentration is highly dependent on the variety and cultivar, with different tomato varieties exhibiting varying responses to changes in anthocyanin levels^11^.

Our study observed the highest leaf glucose, sucrose, and total sugar levels in the control plants under non-irrigated conditions. Conversely, the lowest levels of these sugars were found in plants treated with *Azospirillum* and *Trichoderma* under 100% WR, which could be attributed to the increased water and nutrient absorption facilitated by the biological compounds, which may have influenced sugar metabolism. A similar trend was observed in a study by Vukelić et al.^11^, where two tomato varieties accumulated more starch and soluble sugars (measured in milligrams per gram dry weight) under cold stress conditions compared to control conditions. Interestingly, plants inoculated with *Trichoderma* fungus recorded the lowest sugar levels. This finding suggests that, in our research, plants inoculated with *Trichoderma* managed stress conditions more efficiently. Sugar accumulation serves as an adaptive mechanism to mitigate the effects of decreased osmotic potential under drought stress. Under water-deficient conditions, plants typically increase sugar production to reduce further their internal osmotic potential, which helps prevent severe wilting and enhances water uptake^30^. Additionally, the impact of growth-promoting microorganisms on plant sugar concentration varies depending on the microorganism type and the specific sugar being measured. For example, a study under salinity stress conditions demonstrated that dual inoculation of wheat with *Azospirillum* and *Trichoderma* increased sugar and proline content while reducing sodium accumulation, thus enhancing wheat growth^18^.

In some of the studied traits, the results showed that the combined treatment of *Trichoderma* + *Azospirillum* resulted in weaker outcomes. In certain traits, these results were even lower than those of the control. Although both Trichoderma and Azospirillum are generally considered beneficial for tomato plants, antagonistic interactions may occur under specific conditions. Trichoderma exhibits mycoparasitic activity, competing for resources and producing antifungal compounds, which can potentially inhibit the growth of Azospirillum. Conversely, Azospirillum promotes plant growth through mechanisms that may indirectly affect the efficacy of Trichoderma. The interaction between these microorganisms is complex and influenced by factors such as species specificity, environmental conditions, and the presence of other soil microorganisms^31^. As a known biocontrol agent, Trichoderma can directly parasitize other fungi, potentially including certain Azospirillum species, thereby reducing their population in the rhizosphere. Additionally, both organisms compete for nutrients and space within the soil environment^32^.

In the current study, plant inoculation with *Trichoderma* resulted in the highest fresh fruit and dry weight. Applying biological compounds enhanced the fruit’s fresh and dry weight across all irrigation treatments. Inoculation with beneficial microorganisms provides an effective strategy to reduce fertilizer dependence while improving nutrient absorption and fruit characteristics, as evidenced by the results of this research. Klunklin and Savage^33^ reported significant effects of varying water scarcity levels on fruit quality, including fruit weight, length, and total yield. Additionally, studies have examined the impact of full versus deficit irrigation on tomato plant performance, revealing that reduced irrigation leads to a decrease in fruit size.

The findings of Singh et al.^12^ on tomato fruits align with the results of the current study. In our research, plant inoculation with biological compounds improved fruit firmness and soluble solids across all irrigation treatments. The enhancement of dry matter and drought tolerance resulting from inoculation with PGPR containing ACC-deaminase in porple basil^10^, mung bean^9^ and common chickpea^34^ under stress conditions has been reported. Ullah et al.^5^ isolated an *Azospirillum* strain from tomato rhizosphere and estimated gibberellic acid production to be 3.3 to 5.9 g per 25 mm of liquid culture medium. GA3, a key product of *Azospirillum*, promotes fruit enlargement. However, water potential and oxygen concentration influence GA3 production in A. lipoferum, decreasing production under high water potential and low oxygen conditions. The bacterial population around plant roots is more versatile in nutrients solubilization, transformation, and mobilization. It is carried out by synthesizing diverse arrays of plant hormones. such as auxin, cytokinin (Ck), gibberellin, abscisic acid (ABA), and indole-3-acetic acid (IAA)^35^.

On the other hand, increasing leaf chlorophyll content enhances light absorption by chloroplasts, thereby improving plant assimilation rates. This increase in assimilates can promote leaf surface area expansion, ultimately boosting productivity and contributing to higher fruit and root dry weight^36^. Additionally, elevated chlorophyll levels activate various cellular components, which can significantly enhance enzyme activities involved in fruit ripening, accelerating the ripening process. One of the consequences of ripening is fruit softening. Therefore, increased leaf chlorophyll content during ripening indirectly reduces fruit softness. It also facilitates the movement of nutrients and photosynthetic products to the fruit, lowering its osmotic potential and increasing water absorption in the fruit cells to balance osmotic pressure, diluting the cell sap and reducing soluble solid content. Rahman et al.^37^ support these findings, showing that higher chlorophyll levels correlate with increased ascorbic acid and titratable acidity while reducing soluble solids and fruit pH. The rise in leaf chlorophyll content and assimilate production appears to promote the movement of leaf nutrients to the fruit and storage organs, which may contribute to the observed decrease in leaf glucose, sucrose, and total carbohydrate concentrations.

In contrast, Klunklin and Savage^33^ found that tomatoes’ soluble solid content did not significantly change under drought stress. Similar results were observed when comparing irrigation at 50% and 75% of WR, suggesting that tomatoes’ soluble solids may be less sensitive to mild environmental fluctuations. However, significant changes in soluble solids are likely to occur under more severe environmental stress.

The results showed that the 100% WR had a significant impact on tomato performance. One of the treatments that influenced tomato performance was the *Trichoderma* treatment. It seems that *Trichoderma*, by aiding the tomato roots in absorbing water and nutrients, was able to achieve better results compared to other treatments. Previous studies have also reported that *Trichoderma* plays a crucial role in the development of the plant’s root system. The enhancement of the root system increases the plant’s access to water and nutrients, ultimately leading to improved plant performance. This effect is more evident in the 75% WR treatment combined with *Trichoderma*. In this treatment, despite a 25% reduction in the plant’s water requirements, the performance did not show a significant difference compared to the control treatment under the 100% WR. It seems that *Trichoderma* has supported the tomato plant under stress and water deficit conditions, likely by helping the plant cope with stress and improving water absorption. Other researchers have also reported that *Trichoderma*, more than *Azospirillum*, was able to enhance growth and performance indices in lettuce plants. Furthermore, these researchers reported that the combination of *Trichoderma* and *Azospirillum*, although improving growth, yielded weaker results compared to the individual use of these compounds^23^. One of the potential reasons for the reduced performance in the combined treatments of *Trichoderma* and *Azospirillum* could be complex biological competition, the use of suitable and compatible strains, and the plant’s response to the combined treatment, which requires further investigation. It has also been reported that *Trichoderma* limits the activity of *Azospirillum*^38^.

It appears that *Trichoderma*, through its role in mitigating environmental stresses, increasing endogenous auxin levels and root development, enhancing water and nutrient uptake, improving the rhizosphere, and regulating stomatal structure and function, has more effectively improved WP compared to other treatments. In contrast, *Azospirillum* likely reduced WP due to its stimulation of leaf area expansion and vegetative growth, which may have led to an increased transpiration surface and, consequently, greater water loss. Similar to the present study, previous research on wheat cultivars has also demonstrated that *Trichoderma* treatment improved water use efficiency more effectively than bacterial treatments^16^. Although the precise mechanisms remain unclear, studies on *Arabidopsis* have attributed this improvement to the more effective role of *Trichoderma*, compared to *Azospirillum*, in regulating stomatal activity, thereby enhancing water use efficiency^17^.

## Conclusion

Overall, most plant characteristics, such as fresh and dry root weight and leaf area, improved under the 100% WR, mainly when biological fertilizers, particularly *Trichoderma*, were used. In addition, biochemical characteristics of the plant, such as chlorophyll, had higher levels in the 100% WR along with biological fertilizers. On the other hand, the highest levels of biochemical plant traits such as carotenoids, anthocyanin, glucose, sucrose, and total sugar were obtained in no irrigation cultivation treatment and non-use of biological fertilizers, indicating that the levels of these traits will increase under stressful conditions. Biological fertilizers have supported the plants under stress conditions. However, measured traits in fruits showed that traits like the fresh and dry weight of the fruit, fresh fruit yield and Wp in a 100% WR and the use of biological fertilizers, especially *Trichoderma*, had the highest amount. *Azospirillum* treatment and combining *Trichoderma* with *Azospirillum* could produce the lowest levels of fruit firmness and soluble solid content under a 100% WR. In contrast, the highest levels of the mentioned traits were achieved in the no-irrigation regime. Furthermore, the general evaluation of the results obtained in different WR indicates the effective role of biological fertilizers, especially *Trichoderma*, in stress conditions for tomato plants. In other words, *Trichoderma* and *Azospirillum* have prepared the plants to face stressful conditions by aiding in root system development, water, and nutrient absorption, and facilitating the activation of plant stress response mechanisms. This effect was more pronounced in treatments with *T. harzianum* compared with *A. brasilense* alone. Overall, the results suggest that biological fertilizer application, particularly *Trichoderma*, can enhance tomato physiological performance and tolerance to water stress. Notably, the *Trichoderma* treatment under 75% WR achieved performance comparable to the control treatment under 100% WR, indicating its potential to mitigate the adverse effects of reduced irrigation. Furthermore, under 100% WR conditions, the application of *Trichoderma* was associated with improved yield and crop water productivity (WP), highlighting its role in improving water-use efficiency in tomato production.

## Materials and methods

### Location and experiment design

This experiment was conducted over two growing seasons (2017 and 2019) at the Gorgan University of Agricultural Sciences and Natural Resources experimental field in Golestan Province, Iran, with UTM coordinates E 261,312 and N 4,079,189, S40. The experimental design was factorial based on randomized complete blocks with three replications. The first factor was the irrigation regime at four levels: no irrigation, 50%, 75%, and 100% of the water requirement (WR), and the second factor was the biological fertilizer with four treatments: control, *T. harzianum*, *A. brasilense*, and a combination of *T. harzianum* and *A. brasilense*.The seedlings were transplanted on April 10, 2017, and April 10, 2019. Irrigation treatments were applied two weeks after transplanting, once the seedlings had established in the field. The experiment concluded on July 16. Each experimental plot measured 3.3 × 4 m. The spacing between rows was 1.1 m, and the spacing between plants within a row was 0.4 m, resulting in a planting density of 2.27 plants per square meter. The results of the soil analysis at the experimental site for the years 2017 and 2019 are presented in Table 4. The amount of fertilizers applied during the growing season over the two years of the experiment is presented in Table 5.

**Table 4:** Results of soil analysis at the experimental site in the years 2017 and 2019.

Property	Unit	growing seasons	
		2017	2019
Electrical conductivity	dS·m⁻¹	1.306	1.4
pH	–	8.06	8.04
Organic carbon	\%	1.28	1.15
Nitrogen	\%	0.13	0.14
Phosphorus	mg·kg⁻¹	27.1	29.5
Potassium	mg·kg⁻¹	275	283
Zinc	mg·kg⁻¹	0.89	0.51
Manganese	mg·kg⁻¹	9.68	8.66
Iron	mg·kg⁻¹	4.51	4.00
Copper	mg·kg⁻¹	1.29	1.4
Bulk density	g·cm⁻³	1.7	1.7
Sand	%	35.5	34
Silt	%	31	32
Clay	%	33.5	34

**Table 5:** Fertilizer consumption during the cultivation season in the years 2017 and 2019.

Fertilizer (Kg/ha)	growing seasons	
	2017	2019
Rotten manure	25,600	28,350
Urea	290	250
Ammonium monobasic phosphate	20	20
Potassium sulfate	120	110

### Estimating water requirements (WR) and applying irrigation management

The soil bulk density was determined using Eq. 1. Irrigation treatment was applied by sampling the field soil and measuring the soil moisture at the field capacity and wilting point using Eqs. 2 and 3, respectively. The available water and plants water usable were calculated using Eqs. 4 and 5 ^39^.

1 $$\:{\rho\:}_{b}=\frac{{M}_{s}}{{V}_{t}}$$

2 $$\:{\theta\:}_{v}=\frac{{\rho\:}_{b}\times\:{\theta\:}_{m}}{{\rho\:}_{w}}$$

3 $$\:{\theta\:}_{m}=\frac{{M}_{w}}{{M}_{s}}$$

4 $$\:WC=10Z\left({\rho\:}_{b}\right)\left({\theta\:}_{m}\right)\:and/or\:WC=10Z{\theta\:}_{v}$$

5 $$\:AW={WC}_{FC}-{WC}_{PWP}$$

Where: $\:{\theta\:}_{m}$ -weight soil moisture content, $\:{\theta\:}_{v}$ -volumetric soil moisture content, $\:{\rho\:}_{b}$ -Soil bulk density (g cm^− 3^), $\:{\rho\:}_{w}$ -Water density, AW - available water content, $\:{\theta\:}_{{m}_{PWP}}$- soil moisture content at permanent wilting point, $\:{\:\theta\:}_{{m}_{FC}}$- soil moisture weight content at field capacity point, WC - depth of water in the root zone (mm), $\:{WC}_{PWP}$- moisture content at permanent wilting point (mm), $\:{M}_{s}$- Dry soil weight, $\:{WC}_{FC}$- depth of water at field capacity point (mm), $\:{V}_{t}$- Soil volume.

To calculate the daily reference evapotranspiration, Eq. 6 (the Penman–Monteith equation corrected by FAO, 1998) is used. By multiplying the crop coefficient (Kc) to ETo in different growth stages, the daily crop evapotranspiration is calculated using Eq. 7. The required meteorological and climatic data were obtained from the nearest meteorological station of Golestan Province Meteorological Organization (Hashemabad meteorological station).

6 $$\:{ET}_{o}=\frac{0.408\varDelta\:\left({R}_{n}-G\right)+\gamma\:\frac{900}{T+273}{u}_{2}({e}_{s}-{e}_{a})}{\varDelta\:+\gamma\:(1+0.34{u}_{2})}$$

7 $$\:{ET}_{c}={ET}_{o}\times\:{K}_{c}$$

ETo -Reference Crop Evapotranspiration (mm day^− 1^), $\:\:{R}_{n}$-Net radiation at the crop surface (MJ m^− 2^ day^− 1^), G -Soil heat flow (MJ m^− 2^ day^− 1^), T -Mean daily air temperature (°C), $\:{u}_{2}$ -Wind speed (m s^− 1^), $\:{e}_{s}$ -Saturation vapor pressure (KPa), $\:{e}_{a}$ -Actual vapor pressure (KPa), $\:{e}_{s}-{e}_{a}$ –Saturation vapor pressure deficit (KPa), $\Delta$-Slope of vapor pressure curve (KPa ℃ ^− [1^), $\gamma$Psychrometric constant (KPa ℃^−1^), ETc - Crop Evapotranspiration (mm day^− 1^), $\:{K}_{c}$ -Crop coefficient.

Irrigation treatments were implemented after the establishment of plants in the field and continued until the ripening stage. Given the regional precipitation during the experimental years, tomato cultivation under rain-fed conditions appears feasible. The climatic data and monthly rainfall amounts recorded during the experimental period are provided in Table 6. Result raining data and relative humidity levels indicate that rainfed tomato cultivation is feasible, though lower yields compared to irrigated practices should be anticipated. Irrigation timing was determined based on 50% soil moisture depletion. Specifically, irrigation was applied when the soil moisture level dropped to 40% of the available moisture. The available moisture was calculated by measuring soil moisture at field capacity and permanent wilting points and then determining the difference alongside the root penetration depth. Additionally, the daily moisture uptake by tomato plants was estimated using the Penman-Monteith equation. During irrigation, 50%, 75%, or 100% of the water required to restore soil moisture to agricultural capacity was applied to the respective treatments. Irrigation was carried out using a drip irrigation system, and the irrigation volume was determined based on calculated crop water requirements and measured and applied using a water meter.

**Table 6:** Monthly Climatic data for Gorgan during the growing seasons of 2017and 2019 (Hashemabad synoptic Station).

Growing seasons	Monthe	Minimum temperature	Maximum temperature	Average temperature	Raining (Millimeter)	Average relative humidity (percentage)
		Degrees Celsius				
2017	April	8.5	19.6	14.1	40.4	78
	May	14.3	26.3	20.3	37.1	74
	June	18.4	31.9	25.1	2.2	60
	July	22.5	34.4	28.4	5	57
2019	April	10	14.7	14.7	74.7	82
	May	13.8	20.3	20.3	51.2	67
	June	20.5	26.3	26.3	0.6	58
	July	24	33.2	28.6	39.6	67

### Crop water productivity (WP)

Crop water productivity (WP) measures how much yield is produced per unit of water used. It is an important indicator of agricultural sustainability, reflecting how efficiently water is used to produce tomato fruit. Crop water productivity is derived from yield and crop water use using Eq. 8^40^. Based on the performed calculations, the amount of water use for the treatments is presented in Table 7.

**Table 7:** Water use during the growing season under different irrigation treatments.

Water requirement (WR)	Water use during the growing season (m^3^/ha)	
	2017	2019
100%	17306.48	15989.88
75%	13622.84	12502.59
50%	9939.19	9015.31
no irrigation (just rain)	1922.69	3770.47

8 $$\:WP=\frac{\mathrm{F}\mathrm{r}\mathrm{e}\mathrm{s}\mathrm{h}\:\mathrm{f}\mathrm{r}\mathrm{u}\mathrm{i}\mathrm{t}\:\mathrm{y}\mathrm{i}\mathrm{e}\mathrm{l}\mathrm{d}}{\mathrm{W}\mathrm{a}\mathrm{t}\mathrm{e}\mathrm{r}\:\mathrm{u}\mathrm{s}\mathrm{e}}$$

### Application of biological treatments

To apply *Trichoderma* and *Azospirillum* treatments, tomato seedling roots were soaked in the prepared solutions before planting. The tomato variety Hypeel-303, supplied by Seminis Company (Falat Iran, Bokharest Ave), was used in the experiment. Tomato cultivar Hypeel-303 has a determinate growth habit and is suitable for dual-purpose use, including table and processing. *T. harzianum* was provided as Tricormix-H (10⁶ CFU/mL) from Fanavaran Hayat Sabz Company. Seedling roots were immersed in a 5% *Trichoderma* solution before planting. *A. brasilense* (10⁸ bacteria/mL) was sourced from the Karaj Soil and Water Research Institute. The Azospirillum treatment was applied according to the protocol of the Karaj Soil and Water Research Institute, and the seedling roots were immersed in the supplied solution for 10 min.

### Measurement of traits

At the end of the experiment, 95 days after transplanting, root fresh weight, root dry weight, and leaf area were measured. Belowground root biomass was quantified by excavating roots from a defined soil volume using soil cores or transect-based sampling. Recovered roots were gently washed to remove adhering soil particles, and fresh weight was determined immediately after surface moisture removal. Subsequently, root samples were oven-dried at 60–70 °C to a constant mass and weighed using an analytical balance. This procedure enabled the determination of root fresh weight, root dry weight, and root moisture content^41^.

### Chlorophyll a, chlorophyll b, total chlorophyll, and carotenoids

Leaf samples were collected at the harvest stage of the crop.Chlorophyll a, chlorophyll b, total chlorophyll, and carotenoids were measured using the Arnon (1967)^42^ method; In this method, 0.5 g of fresh tomato (W) leaf tissue was first ground in a porcelain mortar with 10 mL of 80% acetone (80% acetone + 20% distilled water). The resulting mixture was then transferred to a test tube. The test tube containing the acetone-ground leaf mixture was centrifuged at 6000 rpm for 10 min. After centrifugation, the supernatant was transferred to a 50 mL volumetric flask. Subsequently, 10 mL of 80% acetone was added to the residue remaining in the test tube, and the sample was centrifuged again under the same conditions. The supernatant was again transferred to the same volumetric flask. This process was repeated until the total volume of the extract in the flask reached 50 mL.

Once the extract volume reached 50 mL (V), the absorbance of the solution was measured at wavelengths of 480, 510, 645, and 663 nm using a Unico 2800 UV/VIS spectrophotometer. Based on the absorbance values at the specified wavelengths, the concentrations of chlorophyll a (Eq. 9), chlorophyll b (Eq. 10), total chlorophyll (Eq. 11), and carotenoids (Eq. 12) were calculated.

9 $$\:\mathrm{C}\mathrm{h}\mathrm{l}\mathrm{o}\mathrm{r}\mathrm{o}\mathrm{p}\mathrm{h}\mathrm{y}\mathrm{l}\mathrm{l}\:\mathrm{a}\:\mathrm{m}\mathrm{g}/\mathrm{g}\:\mathrm{F}\mathrm{W}=\:\left[12.7\:\right(\mathrm{A}663)\:\--\:2.69\:(\mathrm{A}645\left)\right]\:\mathrm{*}\:\mathrm{V}/\mathrm{W}$$

10 $$\:\mathrm{C}\mathrm{h}\mathrm{l}\mathrm{o}\mathrm{r}\mathrm{o}\mathrm{p}\mathrm{h}\mathrm{y}\mathrm{l}\mathrm{l}\:\mathrm{b}\:\mathrm{m}\mathrm{g}/\mathrm{g}\:\mathrm{F}\mathrm{W}=\:\left[22.9\:\right(\mathrm{A}645)\:\--\:4.68\:(\mathrm{A}663\left)\right]\:\mathrm{*}\:\mathrm{V}/\mathrm{W}$$

11 $$\:\mathrm{C}\mathrm{h}\mathrm{l}\mathrm{o}\mathrm{r}\mathrm{o}\mathrm{p}\mathrm{h}\mathrm{y}\mathrm{l}\mathrm{l}\:\mathrm{t}\mathrm{o}\mathrm{t}\mathrm{a}\mathrm{l}\:\mathrm{m}\mathrm{g}/\mathrm{g}\:\mathrm{F}\mathrm{W}=\:\left[20.2\:\right(\mathrm{A}645)\:\--\:8.02\:(\mathrm{A}663\left)\right]\:\mathrm{*}\:\mathrm{V}/\mathrm{W}$$

12 $$\:\mathrm{C}\mathrm{a}\mathrm{r}\mathrm{o}\mathrm{t}\mathrm{e}\mathrm{n}\mathrm{o}\mathrm{i}\mathrm{d}\mathrm{s}\:\mathrm{m}\mathrm{g}/\mathrm{g}\:\mathrm{F}\mathrm{W}=\:\left[7.6\:\right(\mathrm{A}480)\:\--\:1.49\:(\mathrm{A}510\left)\right]\:\mathrm{*}\:\mathrm{V}/\mathrm{W}$$

### Anthocyanins

Anthocyanins was measured using the Wanger (1979)^43^ method; For this purpose, 1 gram of tomato fruit peel and pulp was thoroughly ground in a porcelain mortar with 10 mL of acidified methanol (99% methanol + 1% hydrochloric acid). Once the fruit tissue was completely homogenized in the acidified methanol, the mixture was transferred to a test tube and kept in darkness at 4 °C for 24 h. After the extraction period, the test tubes containing the extract were centrifuged at 4000 rpm for 10 min. The supernatant, which contains anthocyanins, was then measured at 520 nm using a Unico 2800 UV/VIS spectrophotometer. The anthocyanin content was calculated using Eq. 13.

13 $$\:\mathrm{A}=\:\mathrm{b}\mathrm{c}$$

(A is the absorbance, b is the cuvette path length (1 cm), c is the anthocyanin content expressed as µg. g^[- [1^ of fresh weight, and ε is the molar extinction coefficient, equal to 33,000 mM⁻¹·cm⁻¹).

### Leaf glucose

Leaf samples were collected at the time of harvest to quantify leaf sugar concentrations. leaf glucose was measured using the Miller (1959)^44^ method; For glucose determination, 1.5 mL of the concentrated extract containing soluble sugars was mixed with 1.5 mL of dinitrosalicylic acid (DNS) reagent and incubated in a water bath at 90 °C for 20 min. Immediately after heating, 0.5 mL of 40% potassium sodium tartrate was added to the mixture. After cooling the tubes to room temperature, absorbance was measured at 575 nm using a Unico 2800 UV/VIS spectrophotometer. Glucose content in each sample was then calculated based on a standard calibration curve. Finally, the glucose content was calculated using Eq. 14 derived from the standard calibration curve.

14 $$\:\mathrm{G}\mathrm{l}\mathrm{u}\mathrm{c}\mathrm{o}\mathrm{s}\mathrm{e}\:(\mathrm{m}\mathrm{g}.\mathrm{m}\mathrm{l}-\hspace{0.17em}1)\hspace{0.17em}=\hspace{0.17em}0.836\mathrm{X}-\:0.045$$

(X: the measured absorbance)

### Leaf sucrose

leaf sucrose was measured using the Van Handel (1968)^45^ method; For sucrose determination, 0.1 mL of the concentrated alcoholic extract was mixed with 0.1 mL of 30% potassium hydroxide and incubated in a water bath at 100 °C for 10 min. After cooling the tubes to room temperature, 3 mL of anthrone reagent was added, and the mixture was incubated in a water bath at 40 °C for 20 min. After a second cooling period, absorbance was measured at 620 nm using a Unico 2800 UV/VIS spectrophotometer. Sucrose content of each sample was then calculated using the standard calibration curve. Finally, the sucrose content was calculated using Eq. 15 derived from the standard calibration curve.

15 $$\:\mathrm{S}\mathrm{u}\mathrm{c}\mathrm{r}\mathrm{o}\mathrm{s}\mathrm{e}\:(\mathrm{m}\mathrm{g}.\mathrm{m}\mathrm{l}-\hspace{0.17em}1)\hspace{0.17em}=\hspace{0.17em}0.883\mathrm{X}-\:0.004$$

(X: the measured absorbance)

### Total leaf sugar

Total leaf sugar was measured using the Mccready et al. (1950)^46^ method. In this method, 200 µL of the concentrated extract was mixed with 3 mL of anthrone reagent and incubated in a water bath at 100 °C for 20 min. After cooling, the absorbance of each treatment was measured at 620 nm using a Unico 2800 UV/VIS spectrophotometer. The total sugar content of each sample was then calculated based on the corresponding standard calibration curve. Finally, the total sugar content was calculated using Eq. 16 derived from the standard calibration curve.

16 $$\:\mathrm{T}\mathrm{o}\mathrm{t}\mathrm{a}\mathrm{l}\:\mathrm{l}\mathrm{e}\mathrm{a}\mathrm{f}\:\mathrm{s}\mathrm{u}\mathrm{g}\mathrm{a}\mathrm{r}\:(\mathrm{m}\mathrm{g}.\mathrm{m}\mathrm{l}-\hspace{0.17em}1)\hspace{0.17em}=\hspace{0.17em}0.625\mathrm{X}-0.011$$

(X: the measured absorbance)

Fruit characteristics, including fresh weight, dry weight, firmness, and total soluble solids content, were measured 80 days after transplanting across different treatments. Total soluble solids were assessed using a digital refractometer (Model Neerveld 14-B2550, CETi, Belgium). At harvest, ten fruits were randomly collected per treatment in each replicate, and fruit traits were measured. The mean of the ten fruits was considered as one experimental unit for statistical analysis.

### Data analysis

The Bartlett test was performed to confirm the homogeneity of variances between the experimental years. Subsequently, the data were analyzed using mixed analysis of variance (ANOVA), where year and year interactions were treated as random effects, while all other factors were considered fixed effects. The least significant difference (LSD) test was also used for mean comparisons. Data analysis was performed using SAS 9.4 (SAS Institute, Inc.). Results are presented focusing on the irrigation × biological fertilizer interaction, as it was significant for most traits and adequately represents the combined effects of both factors. The higher-order interaction (year × irrigation × biological fertilizer) was insignificant for any measured trait. Changes in each trait relative to the control treatment (100% WR and no biological fertilizer) were expressed as percentages. Pearson correlation coefficients were calculated based on treatment means to assess relationships among the measured traits.

## Data Availability

The datasets used and/or analysed during the current study are available from the corresponding author on reasonable request.
